# The metabolism of nonstructural carbohydrates, lipids, and energy in two *Cycas* species with differential tolerance to unexpected freezing stress

**DOI:** 10.3389/fpls.2023.1301560

**Published:** 2023-12-07

**Authors:** Yangyang Wu, Huan Zhu, Zhiwei Ling, Erya Lu, Xiaoling Peng, Yanling Zheng

**Affiliations:** Key Laboratory of State Forestry and Grassland Administration for Biodiversity Conservation in Southwest China, Southwest Forestry University, Kunming, Yunnan, China

**Keywords:** energy metabolism, glycerolipids, starch, soluble sugar, unexpected freezing stress

## Abstract

**Introduction:**

With the climate warming, the occurrence of freezing events is projected to increase in late spring and early autumn in the Northern Hemisphere. Observation of morphological traits showed that *Cycas panzhihuaensis* was more tolerant to unexpected freezing stress than *C. bifida*. Energy balance is crucial for plant tolerance to stress. Here, we aimed to determine whether the different responses of the two species to the unpredicted freezing stress were associated with the metabolism of energy and related substances.

**Methods:**

The effects of unexpected freezing temperatures on *C. panzhihuaensis* and *C. bifida* were studied by measuring chlorophyll fluorescence parameters, energy charge and the profile of nonstructural carbohydrates (NSC) and lipids.

**Results:**

*C. panzhihuaensis* exhibited higher stability of photosynthetic machinery than *C. bifida* under unpredicted freezing events. Significant interaction between species and treatments were observed in the energy charge, the level of NSC and its most components and the amount of most lipid categories and lipid classes. The decrease of soluble sugar and the increase of neutral glycerolipids at the early freezing stage, the accumulation of membrane glycerolipids at the late freezing stage and the continuous decrease of energy charge during the freezing period were the characteristics of *C. panzhihuaensis* responding to unexpected freezing stress. The degradation of membrane glycerolipids and the continuous decrease of soluble sugar during the freezing period and the accumulation of neutral glycerolipids and energy charge at the late freezing stage represented the characteristics of *C. bifida* responses.

**Discussion:**

The different freezing sensitivity between *C. panzhihuaensis* and *C. bifida* might be associated with the differential patterns of the metabolism of energy, NSC and lipids. *C. panzhihuaensis* possesses the potential to be introduced to the areas of higher latitudes and altitudes.

## 1 Introduction

Freezing stress can decrease membrane fluidity and the freezing-induced extracellular ice crystals can cause dehydration and physical stress, which result in the collapse of cell structure (Webb et al., 1994; Uemura et al., 1995; Barnes et al., 2016). Photosynthesis is one of the physiological processes that is highly sensitive to cold temperatures (Ensminger et al., 2006). It has been reported that freezing stress can cause the rupture of thylakoid membranes where the photosynthetic apparatus are located (Hincha et al., 1987; Goral et al., 2012). Therefore, photoinhibition and photodamage might occur to various extents depending on plant sensitivity to freezing temperatures (Cavender-Bares, 2007). The precise control of energy homeostasis that depends on the coordination of photosynthesis, photorespiration, and respiration is crucial for plant development and tolerance to stress conditions (Dröge-Laser and Weiste, 2018; Shameer et al., 2019). However, low temperatures can induce an imbalance between the light energy absorbed by photosystems and the energy consumed by metabolic sinks (Huner et al., 1996; Ensminger et al., 2006). Correspondingly, the metabolism of carbohydrates and lipids that represent two major forms of energy storage in plants is likely to be regulated by freezing stress. However, how plants regulate the metabolism of energy, carbohydrates, and lipids under freezing stress is poorly understood.

The dynamics of nonstructural carbohydrates (NSCs) including starch and soluble sugars are key determinent of plant growth, productivity, and resistance ability under environmental stress (Dietze et al., 2014; Martínez-Vilalta et al., 2016; Thalmann and Santelia, 2017). Starch is a major energy storage polysaccharide in most plants, and soluble sugars can serve as osmolytes, antioxidants, and signaling molecules (Couée et al., 2006; Stitt and Zeeman, 2012; Vitova et al., 2015; Saddhe et al., 2021). Soluble sugars can decrease the cytoplasmic freezing point and prevent the cellular dehydration induced by the intercellular ice formation (Burg and Ferraris, 2008; Ouellet and Charron, 2013). The starch degradation and accumulation of soluble sugars is involved in the acquisition of freezing tolerance for some plants (Yano et al., 2005; Thalmann and Santelia, 2017). Zareei et al. (2021) showed that the dynamic change of soluble sugars with the decrease of freezing temperatures (−5°C to −25°C) was dependent on cultivars and plant organs. The carbohydrate concentration of *Pinus brutia* also dynamically changed with the decrease of freezing temperatures (−5°C to −20°C), which was related to the provenances and growth stages (Yildiz et al., 2014).

Lipids are used not only as energy sources but also as principal constituents of biological membranes and signaling molecules in plants (Hou et al., 2016; Yang and Benning, 2018; Chng et al., 2022). Membrane is the major site of damage induced by low temperatures in plants (Hincha et al., 1987). For some plants, freezing stress can lead to the degradation of the membrane lipids including phospholipids and saccharolipids (Zheng et al., 2016). However, some other plants can remodel the membrane lipids to survive the low temperatures (Thomashow, 1999). Studies have shown that the levels of plastidic and extraplastidic membrane lipids and the unsaturation of these lipids can increase under chilling and freezing conditions to maintain the integrity and fluidity of membranes (Welti et al., 2002; Zheng et al., 2021). In general, the accumulation of the bilayer-stabilizing lipids such as phosphatidylcholine (PC), digalactosyldiacylglycerol (DGDG), and oligogalactolipids is conducive to the acquirement of freezing tolerance (Moellering et al., 2010; Zhang et al., 2013; Arisz et al., 2018).

For temperate plants, many species can acquire freezing tolerance through a period of cold acclimation at low but non-freezing temperatures (Chang et al., 2021). In addition, subsequent exposure to moderate freezing temperatures can further enhance freezing tolerance (Junttila and Kaurin, 1990; Herman et al., 2006). However, due to the lengthening of the growing season of plants induced by climate warming, the freezing events occurring in early autumn and late spring are likely to increase in some areas of the Northern Hemisphere (Liu et al., 2018; Zohner et al., 2020). The unpredicted freezing events might have devastating effects on the growth and survival for the plants unprepared for the changing environments (Seydel et al., 2022). For the plants distributed in the tropical and subtropical areas, some of them are vulnerable to low temperatures above 0°C, but some species can be introduced to the higher latitude areas and survive the freezing temperatures there (Huang, 2011). However, very little is known about the response of tropical and subtropical plants to unexpected freezing stress.

*Cycas* species possess high ornamental and scientific research values, which are mainly distributed in tropical and subtropical areas (Hoffmann et al., 2010). Among these species, *C. panzhihuaensis* is limited to the dry-hot valleys of the Jinsha River in southwest China where the northern latitudinal limits of *Cycas* species are observed. The population of this species in Panzhihua National Nature Reserve is well conserved, but other wild populations are very small (Yu et al., 2007). *C. bifida* is restricted to some areas of Yunnan and Guangxi provinces, with extremely small populations (Yin, 2013). Despite the different distribution, both the species can experience more than 40°C in their habitats. Studies have shown that the continuing climate warming will pose higher threat to tropical species than the species distributed in the areas of higher latitude (Doughty and Goulden, 2008; Sentinella et al., 2020). Considering the narrow geographic distributions and occurrence of extreme high temperatures, *Cycas* species might be introduced into areas with lower temperatures to avoid the potential risk of endangerment. However, how these plants including C. *panzhihuaensis* and *C. bifida* respond to the unpredicted freezing stress is poorly understood. On the basis of the observation of morphological traits, *C. panzhihuaensis* was more tolerant to unexpected freezing shock than *C. bifida*. We speculated that this difference between *C. panzhihuaensis* and *C. bifida* might be related to the metabolism of energy, NSC, and lipids. To explore the effects of unexpected freezing stress on the metabolism of energy and related substances and provide theoretical basis for the introduction, the energy status, the photosynthetic activity, NSC concentration, and lipid profiles were determined in the two species.

## 2 Materials and methods

### 2.1 Plant material and experimental procedures

Six-year-old plants of *C. panzhihuaensis* and *C. bifida* were cultivated in a 1:1:1 (v/v/v) mixture of sand, humus, and laterite soil in a greenhouse where the temperature and light intensity cannot be automatically controlled. The temperature was about 15°C to 25°C, and the daytime maximum photosynthetic photon flux density was approximately 600 μmol m^−2^ s^−1^ during the period that we conducted the experiment. The different intensity of freezing stress is achieved by different subzero temperatures with the same treatment duration or achieved by the same subzero temperature with different treatment durations (Lu et al., 2019; Zareei et al., 2021). The temperature and duration of freezing stress were chosen on the basis of our preliminary study. The application of freezing temperature was conducted referring to the procedure described by Arisz et al. (2018) with several modifications. Freezing treatment was conducted in the light (300 μmol m^−2^ s^−1^) by lowering the temperature from 4°C to −1°C at a rate of 2°C h^−1^. After 1 h at −1°C, ice crystals were sprinkled to induce freezing and prevent supercooling. Temperature was then decreased at the same rate to −5°C and maintained for 1.5 h (F1) and 6 h (F2), respectively. Plants were sampled immediately after freezing treatment for measurement of some metabolites. Plants that were not treated at freezing temperatures were used as the controls. To evaluate the freezing tolerance, plants previously subjected to F1 and F2 treatments were transferred to 4°C and thawed for 12 h in the dark and then placed back to the control conditions for 3 days. The chlorophyll fluorescence parameters were measured for both the thawed and recovered plants, and morphological traits of leaves were observed for the recovered plants.

### 2.2 Chlorophyll fluorescence measurement

The plants that were subjected to freezing-thawing stress and subsequent 3 days of recovery were used to measure chlorophyll fluorescence parameters. Measurements were taken after a 30-min dark adaptation of plants at room temperature. Chlorophyll fluorescence was measured in leaves of five plants per treatment with a PAM-2500 portable chlorophyll fluorometer (Walz, Germany). The saturating light pulse was set at about 8,000 µmol m^–2^ s^–1^ for 0.8 s. The actinic light was set at 617 µmol m^–2^ s^–1^, and the plants were adapted to such actinic light for more than 5 min. All the chlorophyll fluorescence parameters were given automatically by the instrument.

### 2.3 Determination of energy substances and energy charge

After being frozen in liquid nitrogen, leaf sample was ground and mixed with cold 1:2:2 (v/v/v) H_2_O/methanol/acetonitrile. The mixture was centrifuged at 4°C for 20 min (14,000g) and dried in a vacuum centrifuge. The samples were dissolved in 1:1 (v/v) acetonitrile/water and centrifuged again at 4°C for 15 min. The supernatants were used for determination of adenosine monophosphate (AMP), adenosine diphosphate (ADP), and adenosine triphosphate (ATP) by liquid chromatography tandem mass spectrometry (LC-MS/MS) analysis using an ultra high performance liquid chromatography (UHPLC) (1290 Infinity LC, Agilent Technologies) coupled to a QTRAP (AB Sciex 5500). Multiquant software was used for data acquisition and processing. Energy charge (EC) was calculated as follows: EC = (ATP + 0.5 × ADP)/(ATP + ADP + AMP).

### 2.4 Determination of nonstructural carbohydrate amount

The concentration of soluble sugars was measured using the protocols described by Salvador et al. (2000) and Wang et al. (2018). Briefly, following the hydrolysis in trifluoroacetic acid (2 M), the leaf sample was dried with nitrogen and then washed two to three times with methanol. Thereafter, the residue was dissolved in deionized water and filtered for measurement. The soluble sugar composition and content of the sample extracts were determined by high-performance anion-exchange chromatography. The starch content was measured using a starch assay kit (Comin, China). The concentration of soluble sugars was calculated by summing the concentrations of each composition of soluble sugars. The concentration of NSC was calculated by summing the concentrations of soluble sugars and starch.

### 2.5 Lipid profiling analysis

The protocols of lipid extraction and detection were described by Zhu et al. (2019). The freeze-dried samples were ground, mixed with 200 µL of cold water and 800 µL of cold methyl tert-butyl ether, and vortexed for 30 s. Thereafter, the mixture was added to 240 µL of methanol, sonicated at 4°C for 20 min and stand for 30 min, and then centrifuged at 14,000g for 15 min at 10°C to extract lipids. The upper organic layer was dried in a vacuum centrifuge. Lipid extracts were resuspended in 200 µL of isopropanol acetonitrile 9:1 (v/v) before LC-MS analysis. For untargeted lipidomics analysis, lipids were separated on a Waters ACQUITY PREMIER CSH C18 Column (1.7 µm, 2.1 mm × 100 mm). MS detection was performed using a Thermo Scientific™ Q Exactive mass spectrometer, equipped with an electrospray ionization (ESI) ion source. Lipidsearch 4.0 software was used for peak detection and annotation of lipids or internal standards. The main parameters are as follows: precursor tolerance, 5 ppm; product tolerance, 5 ppm; and product ion threshold, 5%.

### 2.6 Statistical analysis

Five replicates for each measurement were arranged for each treatment. For measurement of chlorophyll fluorescence, three leaves from each plant were measured, and the average of these values represented the respective plant. For analysis of lipidomics, the amount of NSCs and energy substances, sample collection was performed from five plants for each replicate. All statistical analyses were performed with SPSS 15.0 (IBM Corp., Armonk, NY). To analyze the significance (P ≤ 0.05) of species, treatment and their interactive effects, the data were assessed using two-way analysis of variance (ANOVA). To evaluate the significance of difference among treatments within one species, the data were assessed using one-way ANOVA. To evaluate the significance of difference between species within one treatment, the data were evaluated by the independent sample T-test.

## 3 Results

### 3.1 *C. panzhihuaensis* was more tolerant to unexpected freezing stress than *C. bifida*

For *C. panzhihuaensis*, all the chlorophyll fluorescence parameters except the non-regulated energy dissipation of photosystem II (PSII)–[Y(NO)] maintained unchanged immediately following freezing stress (**Table 1**). Y(NO) of F2-treated plants increased significantly compared to that of the control but recovered to the control level after 3 days of recovery. For *C. bifida*, compared to those of the non-treated plants, the potential quantum yield of PSII (Fv/Fm), effective quantum yield of PSII [Y(II)], photochemical quenching coefficient (qP), and the relative electron transport rate (rETR) declined by 4.7%, 41.3%, 46.8%, and 42.0%, respectively, after F1 treatment and decreased by 22.8%, 83.7%, 84.9%, and 84.7%, respectively, after F2 treatment. Following 3 days of recovery, Fv/Fm, Y(II), qP, and rETR increased significantly in F1-treated plants but decreased significantly in F2-treated plants in comparison with those of plants sampled immediately from F1 and F2 treatments, respectively. Compared to the control, the photoprotective regulated energy dissipation of PSII [Y(NPQ)] increased significantly after F1 treatment but decreased significantly after F2 treatment. After 3 days of recovery, Y(NPQ) maintained in F1-treated plants and further decreased significantly in F2-treated plants. Compared to that of the control, Y(NO) increased significantly following F1 and F2 treatments, by 22.1% and 216.8%, respectively. This parameter recovered to the control level in F1-treated plants but continued to increase significantly in F2-treated plants following recovery. Compared to that of the control, the non-photochemical quenching coefficient (NPQ) remained unchanged in F1-treated plants but decreased significantly by 96.4% in F2-treated plants. Compared to that of the corresponding freezing-treated plants, NPQ increased significantly in F1-treated plants and did not change significantly in F2-treated plants after recovery. Significant interaction was observed between species and treatment on all the chlorophyll fluorescence parameters. These results showed that photosynthetic machinery of *C. panzhihuaensis* was more stable than that of *C. bifida* under unexpected freezing stress. After 3 days of recovery, the leaf morphological traits did not change visibly for all the freezing-treated *C. panzhihuaensis* and F1-treated *C. bifida* (**Supplementary Figure S1**). However, the leaves of F2-treated *C. bifida* plants turned yellow and curled up after recovery.

**Table 1:** The chlorophyll fluorescence parameters of *Cycas panzhihuaensis* and *C. bifida* exposed to unexpected freezing stress and subsequent 3 days of recovery.

	Fv/Fm	Y(II)	Y(NPQ)	Y(NO)	NPQ	qP	rETR
*C. panzhihuaensis*							
Control	0.763 ± 0.002a	0.272 ± 0.031a*	0.442 ± 0.033a*	0.287 ± 0.006b	1.543 ± 0.132ab	0.486 ± 0.042a*	67.750 ± 7.632a*
F1	0.758 ± 0.025a*	0.294 ± 0.022a*	0.389 ± 0.041a*	0.316 ± 0.031ab	1.249 ± 0.238b	0.504 ± 0.028a*	73.667 ± 6.282a*
F2	0.748 ± 0.027a*	0.293 ± 0.030a*	0.377 ± 0.042a*	0.331 ± 0.023a*	1.149 ± 0.191b*	0.556 ± 0.046a*	72.333 ± 7.024a*
F1R	0.781 ± 0.016a	0.309 ± 0.039a*	0.441 ± 0.073a	0.249 ± 0.036b	1.821 ± 0.529a	0.558 ± 0.049a*	75.000 ± 9.000a*
F2R	0.779 ± 0.003a*	0.296 ± 0.019a*	0.454 ± 0.014a*	0.250 ± 0.016b*	1.820 ± 0.137a*	0.533 ± 0.036a*	70.667 ± 4.041a*
*C. bifida*							
Control	0.762 ± 0.008a	0.332 ± 0.020a	0.383 ± 0.030b	0.285 ± 0.012d	1.350 ± 0.158b	0.624 ± 0.027a	83.333 ± 4.502a
F1	0.726 ± 0.011b	0.195 ± 0.013c	0.457 ± 0.020a	0.348 ± 0.010c	1.316 ± 0.086b	0.332 ± 0.020c	48.333 ± 3.055c
F2	0.588 ± 0.024c	0.054 ± 0.007d	0.043 ± 0.017c	0.903 ± 0.022b	0.048 ± 0.020c	0.094 ± 0.016d	12.750 ± 1.389d
F1R	0.764 ± 0.003a	0.254 ± 0.022b	0.463 ± 0.021a	0.284 ± 0.003d	1.632 ± 0.075a	0.426 ± 0.029b	61.800 ± 5.263b
F2R	0.060 ± 0.029d	0.001 ± 0.000e	0.009 ± 0.001d	0.991 ± 0.015a	0.011 ± 0.003c	0.008 ± 0.002e	0 ± 0e
Significance							
Treatment	*	*	*	*	*	*	*
Species	*	*	*	*	*	*	*
Treatment × species	*	*	*	*	*	*	*

Two-way ANOVA analysis was performed in the general linear model. * indicates P ≤ 0.05. For the same species, different letters in the same column are significantly different between treatments according to one-way ANOVA at P ≤ 0.05. For the same treatment, * is significantly different between species according to independent samples T-test at P ≤ 0.05. Values shown are the mean ± SD, n = 5. F1, freezing treatment at −5°C for 1.5 h; F2, freezing treatment at −5°C for 6 h; F1R, 3 days of recovery under control conditions (25°C/15°C) following F1 treatment; F2R, 3 days of recovery under control conditions following F2 treatment; Fv/Fm, the maximum quantum yield of photosystem II (PSII); NPQ, non-photochemical quenching coefficient; qP, photochemical quenching coefficient; rETR, relative electron transport rate; Y(II), effective quantum yield of PS II; Y(NO), non-regulated non-photochemical energy loss in PS II; Y(NPQ), regulated non-photochemical energy loss in PS II.

### 3.2 The two *Cycas* species differentially responded to unexpected freezing stress in metabolism of energy and related substances

#### 3.2.1 The energy status

**Figure 1:**
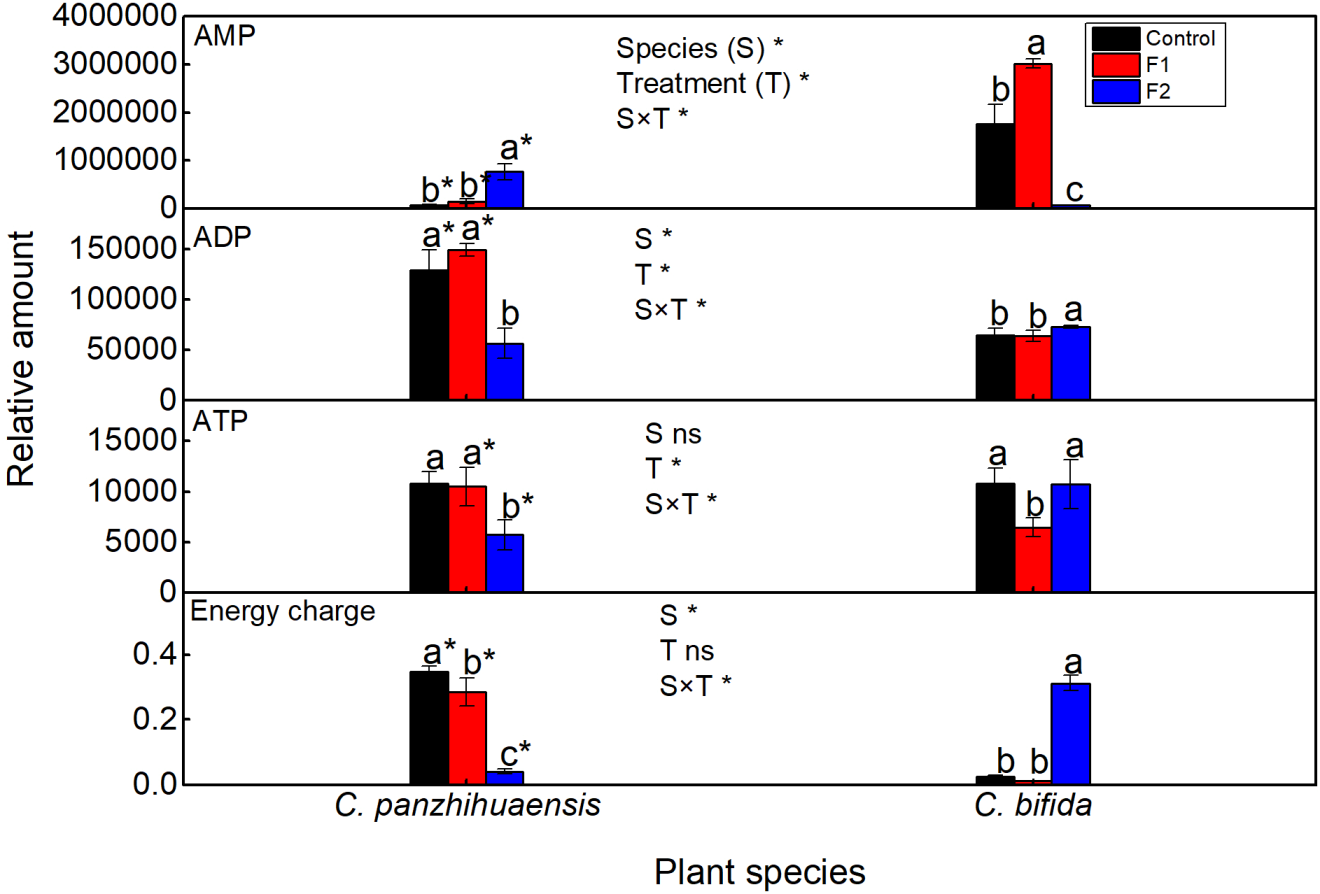
The relative level of adenosine monophosphate (AMP), adenosine diphosphate (ADP), adenosine triphosphate (ATP) and energy charge of *Cycas panzhihuaensis* and *C. bifida* exposed to unexpected freezing stress. Two-way ANOVA analysis was performed in the general linear model. * indicates *P* ≤ 0.05. ns indicates not significant. For the same species, different letters in the same column are significantly different among treatments according to One-way ANOVA at *P* ≤ 0.05. For the same treatment, * is significantly different between species according to independent samples T-test at P ≤ 0.05. Values shown are the mean ± SD, *n* = 5. F1, freezing treatment at -5 °C for 1.5 h; F2, freezing treatment at -5 °C for 6 h.

Compared to those of the control, the relative amount of AMP increased significantly but that of ADP and ATP decreased significantly in F2-treated *C. panzhihuaensis* (**Figure 1**). The energy charge of *C. panzhihuaensis* significantly declined with the duration of freezing treatment. For *C. bifida*, AMP, ADP, and ATP presented different change trend during freezing treatment. When compared to those in the control, the relative amount of AMP increased significantly in F1-treated plants and decreased significantly in F2-treated plants; ADP significantly increased in F2-treated plants, and ATP significantly declined in F1-treated plants. The energy charge remained unchanged in F1-treated plants but increased significantly in F2-treated plants. Significant interaction was observed between species and treatment on the relative amounts of AMP, ADP, ATP, and energy charge.

#### 3.2.2 The level of NSC and its components

**Figure 2:**
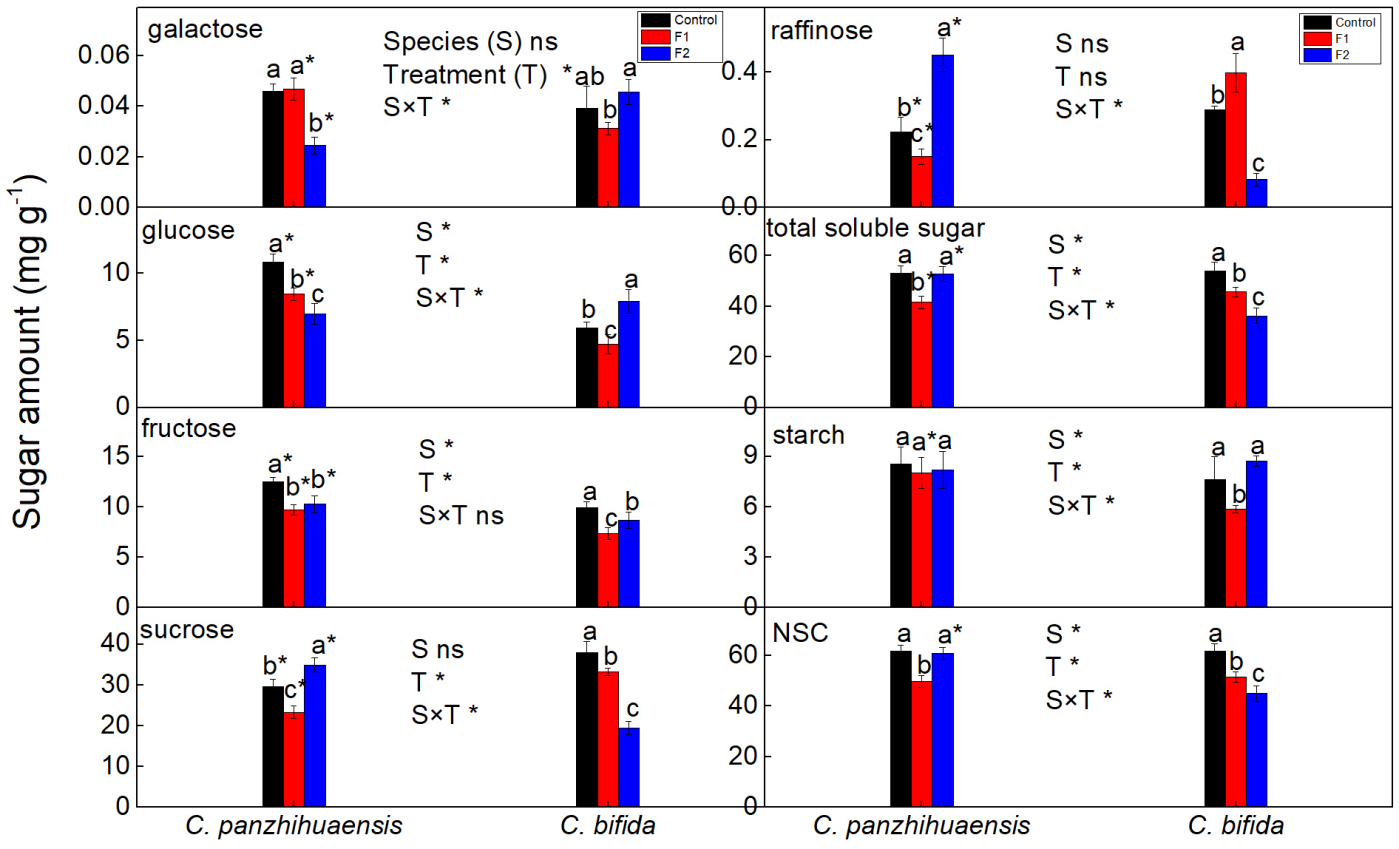
The content of the nonstructural carbohydrates of *Cycas panzhihuaensis* and *C. bifida* exposed to unexpected freezing stress. Two-way ANOVA analysis was performed in the general linear model. * indicates *P* ≤ 0.05. ns indicates not significant. For the same species, different letters in the same column are significantly different among treatments according to One-way ANOVA at *P* ≤ 0.05. For the same treatment, * is significantly different between species according to independent samples T-test at *P* ≤ 0.05. Values shown are the mean ± SD, *n* = 5. F1, freezing treatment at -5 °C for 1.5 h; F2, freezing treatment at -5 °C for 6 h.

For *C. panzhihuaensis*, compared to that of the corresponding control, the amount of glucose, fructose, sucrose, raffinose, and the total soluble sugars decreased significantly following F1 treatment (**Figure 2**). F2 treatment induced the significant decrease of galactose, glucose, and fructose and significant increase of sucrose and raffinose but did not change the total level of soluble sugars. Starch amount remained unchanged following both F1 and F2 treatments. NSC level significantly declined, by 19.6% in F1-treated plants and then increased to the original level in F2-treated plants. For *C. bifida*, the amount of glucose, fructose, sucrose, and the total soluble sugars decreased significantly, and raffinose level significantly increased in F1-treated plants, compared to the control. However, the level of glucose increased significantly and that of fructose, sucrose, raffinose, and the total soluble sugars decreased significantly following F2 treatment, compared to the control. During the freezing period, the starch amount significantly declined, by 23.0% in the early stage and then recovered to the control plant level in the late stage. Compared to that of the non-treated plants, the NSC amount decreased significantly in F1-treated and F2-treated plants, by 16.4% and 27.1%, respectively. Significant interaction was observed between species and treatment on the levels of all the components of NSC except fructose.

#### 3.2.3 The amount and composition of lipids

**Figure 3:**
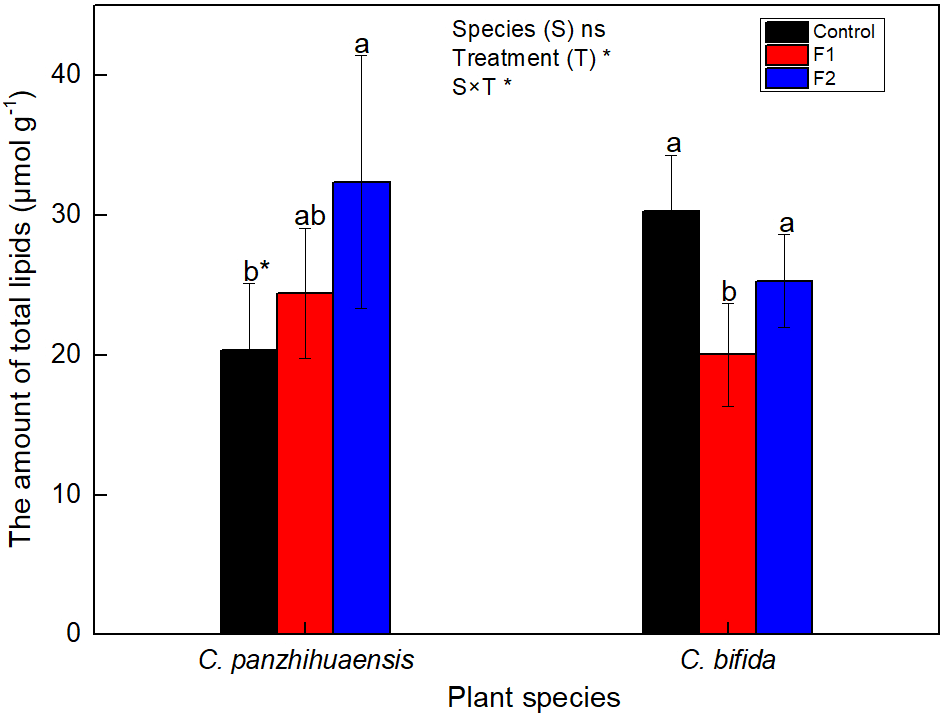
The content of the total lipids of *Cycas panzhihuaensis* and *C. bifida* exposed to unexpected freezing stress. Two-way ANOVA analysis was performed in the general linear model. * indicates *P* ≤ 0.05. ns indicates not significant. For the same species, different letters in the same column are significantly different among treatments according to One-way ANOVA at *P* ≤ 0.05. For the same treatment, * is significantly different between species according to independent samples T-test at *P* ≤ 0.05. Values shown are the mean ± SD, *n* = 5. F1, freezing treatment at -5 °C for 1.5 h; F2, freezing treatment at -5 °C for 6 h.

For *C. panzhihuaensis*, when compared to that of the non-treated plants, the amount of the total lipid increased with the duration of freezing, by 20.0% and 59.2%, respectively, after F1 and F2 treatments (**Figure 3**). For *C. bifida*, the total lipid level decreased significantly, by 33.80% in F1-treated plants, but then increased to the control level in F2-treated plants. Significant interaction was observed between species and treatment on the amount of the total lipids.

**Figure 4:**
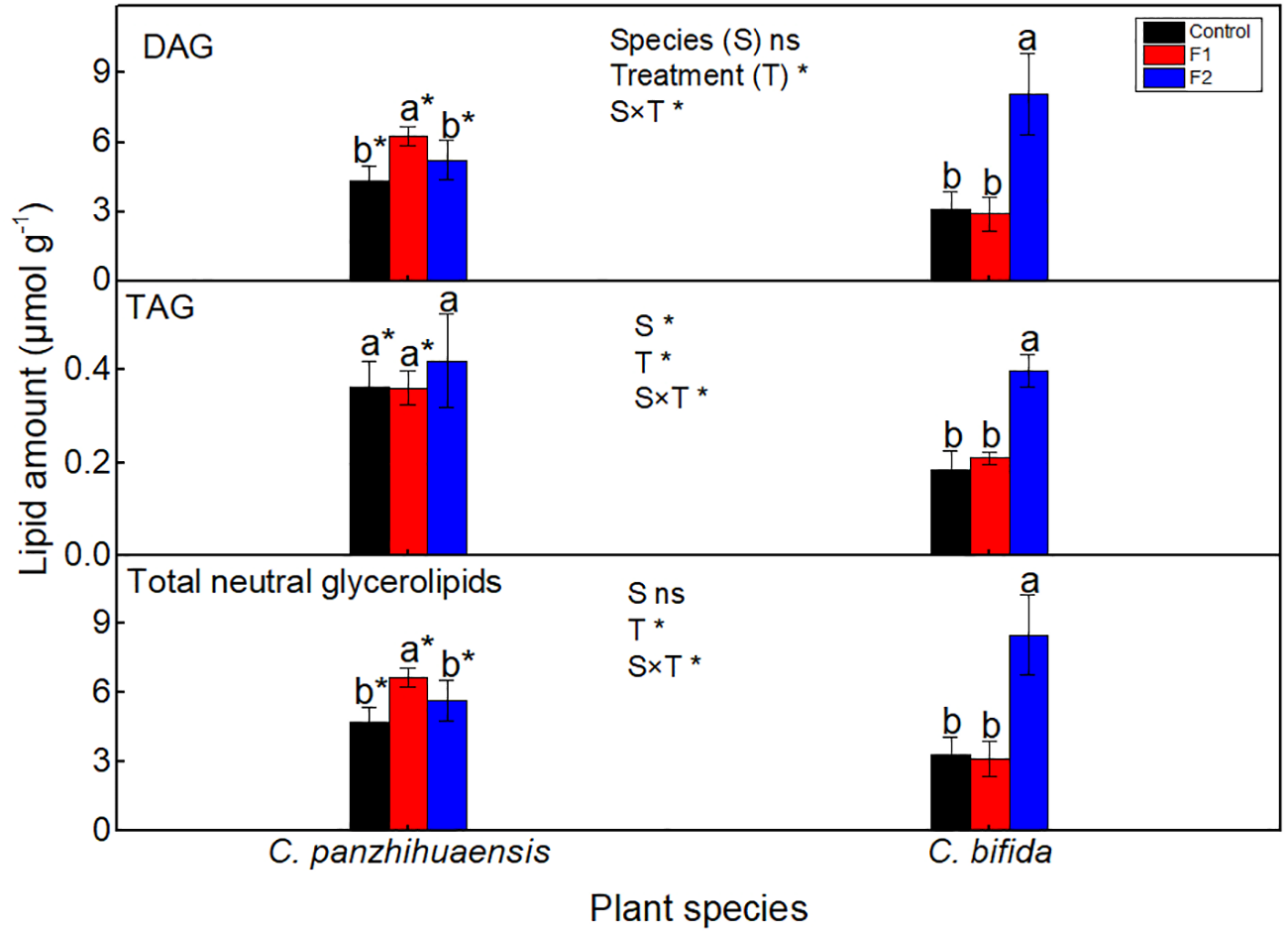
The content of the neutral glycerolipids of *Cycas panzhihuaensis* and *C. bifida* exposed to unexpected freezing stress. Two-way ANOVA analysis was performed in the general linear model. * indicates *P* ≤ 0.05. ns indicates not significant. For the same species, different letters in the same column are significantly different among treatments according to One-way ANOVA at *P* ≤ 0.05. For the same treatment, * is significantly different between species according to independent samples T-test at *P* ≤ 0.05. Values shown are the mean ± SD, *n* = 5. DAG, diacylglycerol; TAG, triacylglycerol; F1, freezing treatment at -5 °C for 1.5 h; F2, freezing treatment at -5 °C for 6 h.

For *C. panzhihuaensis*, the amount of DAG and the total neutral glycerolipids increased by 45.3% and 41.70%, respectively, after F1 treatment, which then recovered to the control level after F2 treatment (**Figure 4**). Freezing stress did not induce significant change of the TAG amount. For *C. bifida*, compared to those of the non-treated plants, the amounts of DAG, TAG, and the total neutral glycerolipids remained unchanged in F1-treated plants but increased significantly in F2-treated plants, by 161.0%, 114.9%, and 158.4%, respectively. Significant interaction was observed between species and treatment on the amounts of DAG, TAG, and the total neutral glycerolipids.

**Figure 5:**
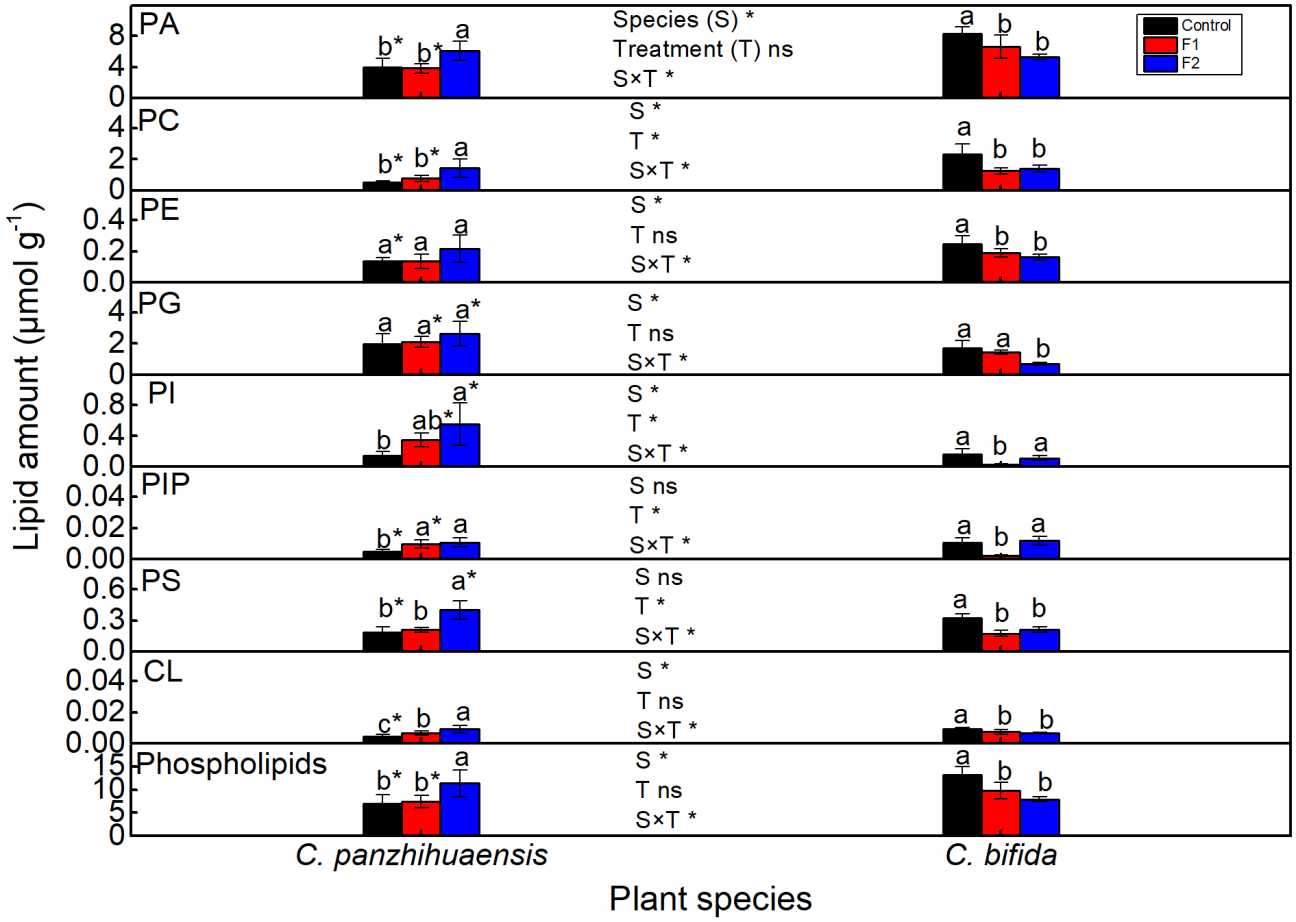
The content of the phospholipids of *Cycas panzhihuaensis* and *C. bifida* exposed to unexpected freezing stress. Two-way ANOVA analysis was performed in the general linear model. * indicates *P* ≤ 0.05. ns indicates not significant. For the same species, different letters in the same column are significantly different among treatments according to One-way ANOVA at *P* ≤ 0.05. For the same treatment, * is significantly different between species according to independent samples T-test at *P* ≤ 0.05. Values shown are the mean ± SD, *n* = 5. F1, freezing treatment at -5 °C for 1.5 h; F2, freezing treatment at -5 °C for 6 h; PA, phosphatidic acid; PC, phosphatidylcholine; PE, phosphatidylethanolamine; PG, phosphatidylglycerol; PI, phosphatidylinositol; PIP, phosphatidylinositol phosphate; PS, phosphatidylserine; CL, cardiolipin.

For *C. panzhihuaensis*, compared to those of the control, phosphatidylinositol (PI) phosphate (PIP) and cardiolipin (CL) increased significantly in F1-treated plants, and the total phospholipids and all the phospholipid classes except phosphatidylethanolamine (PE) and phosphatidylglycerol (PG) increased significantly in amount after F2 treatment (**Figure 5**). For *C. bifida*, the levels of phosphatidic acid (PA), PC, PE, phosphatidylserine (PS), CL, and the total phospholipids decreased significantly following both F1 and F2 treatments. Compared to that of the control, PG amount remained unchanged after F1 treatment and decreased significantly after F2 treatment and the amount of PI and PIP decreased significantly after F1 treatment and increased up to the control level after F2 treatment. Significant interaction was observed between species and treatment on the amounts of each component of phospholipids and the total phospholipids.

**Figure 6:**
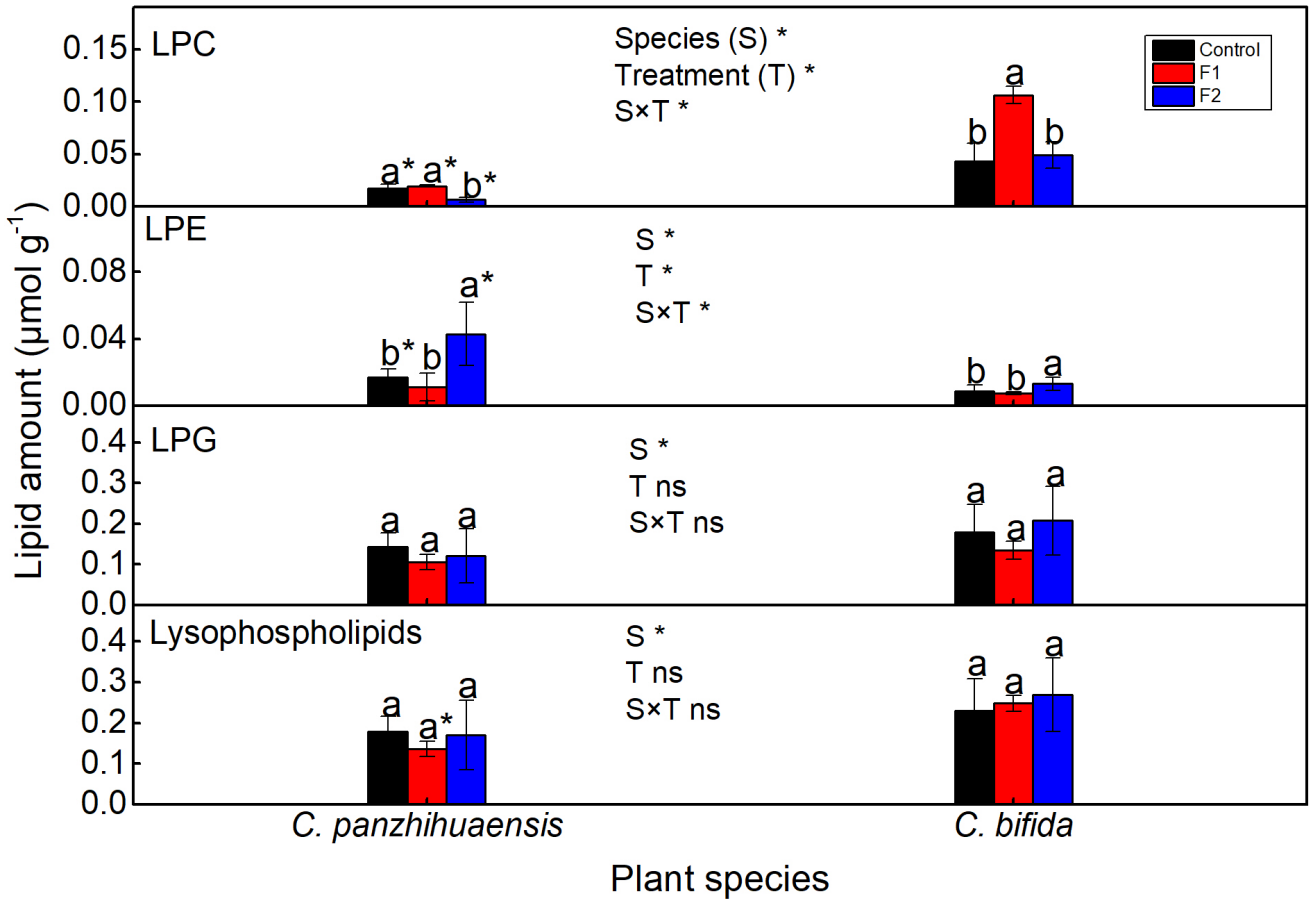
The content of the lysophospholipids of *Cycas panzhihuaensis* and *C. bifida* exposed to unexpected freezing stress. Two-way ANOVA analysis was performed in the general linear model. * indicates *P* ≤ 0.05. ns indicates not significant. For the same species, different letters in the same column are significantly different among treatments according to One-way ANOVA at *P* ≤ 0.05. For the same treatment, * is significantly different between species according to independent samples T-test at *P* ≤ 0.05. Values shown are the mean ± SD, *n* = 5. F1, freezing treatment at -5 °C for 1.5 h; F2, freezing treatment at -5 °C for 6 h; LPC, lysophosphatidylcholine; LPE, lysophosphatidylethanolamine; LPG, lysophosphatidylglycerol.

For *C. panzhihuaensis*, F2 treatment induced the significant decline of lysophosphatidylcholine (LPC) amount and significant increase of lysophosphatidylethanolamine (LPE) amount (**Figure 6**). For *C. bifida*, LPC amount significantly increased in F1-treated plants and LPE significantly increased in F2-treated plants. The amounts of lysophosphatidylglycerol (LPG) and the total lysophospholipids remained unchanged following both F1 and F2 treatments in the two species. Significant interaction was observed between species and treatment on the levels of LPC and LPE.

**Figure 7:**
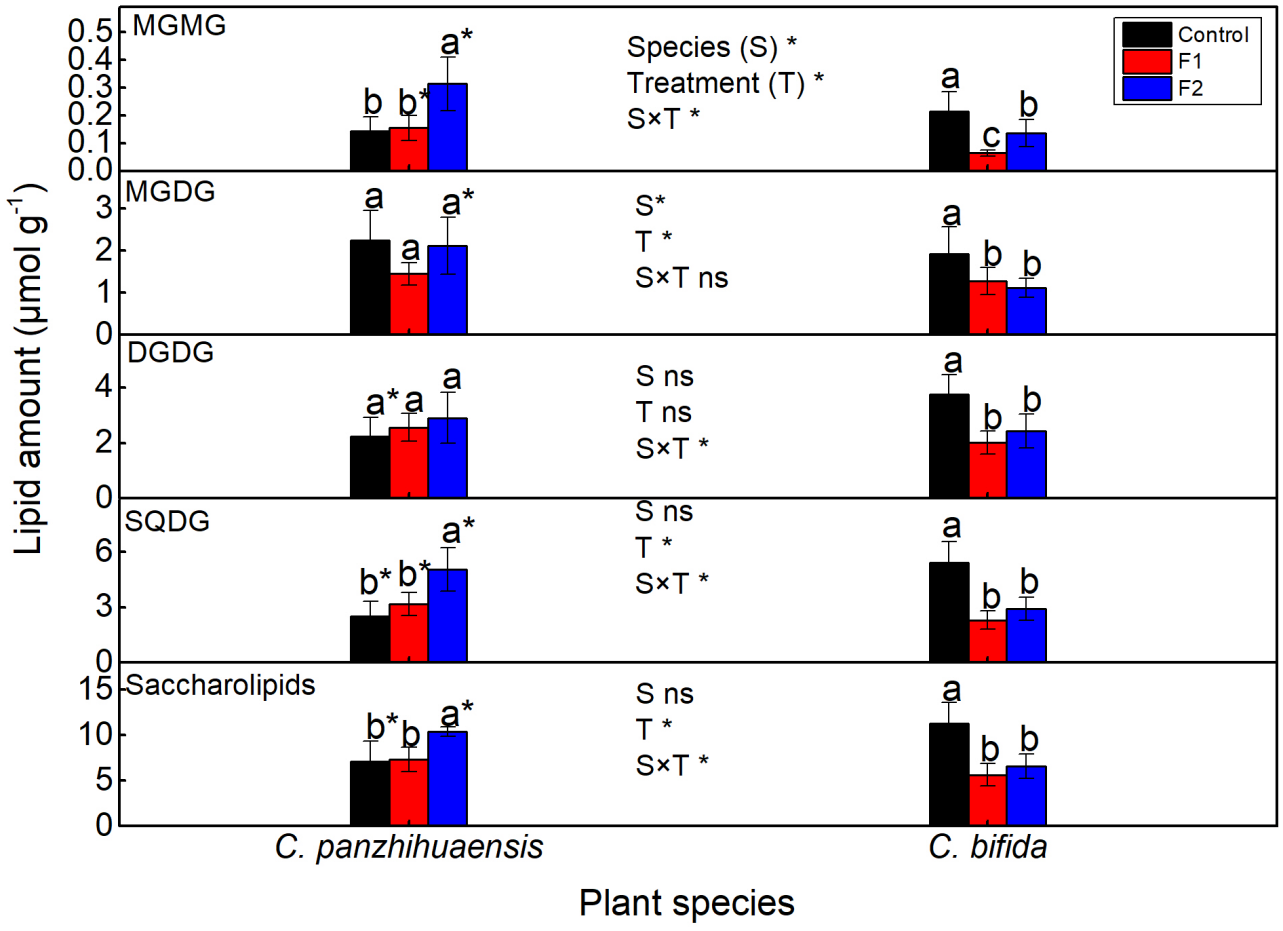
The content of the saccharolipids of *Cycas panzhihuaensis* and *C. bifida* exposed to unexpected freezing stress. Two-way ANOVA analysis was performed in the general linear model. * indicates *P* ≤ 0.05. ns indicates not significant. For the same species, different letters in the same column are significantly different among treatments according to One-way ANOVA at *P* ≤ 0.05. For the same treatment, * is significantly different between species according to independent samples T-test at *P* ≤ 0.05. Values shown are the mean ± SD, *n* = 5. DGDG, digalactosyldiacylglycerol; F1, freezing treatment at -5 °C for 1.5 h; F2, freezing treatment at -5 °C for 6 h; MGDG, monogalactosyldiacylglycerol; MGMG, monogalactosylmonoacylglycerol; SQDG, sulphoquinovosyldiacylglycerol.

Freezing stress did not change the amounts of monogalactosyldiacylglycerol (MGDG) and DGDG of *C. panzhihuaensis* (**Figure 7**). Compared to those in the control group, the levels of monogalactosylmonoacylglycerol (MGMG) and sulphoquinovosyldiacylglycerol (SQDG) and the total saccharolipids remained unchanged in F1-treated plants but significantly increased in F2-treated plants. For *C. bifida*, the level of the total saccharolipids and its each lipid class decreased significantly following both F1 and F2 treatments. Significant interaction was observed between species and treatment on the level of MGMG, DGDG, and SQDG and the total saccharolipids.

**Figure 8:**
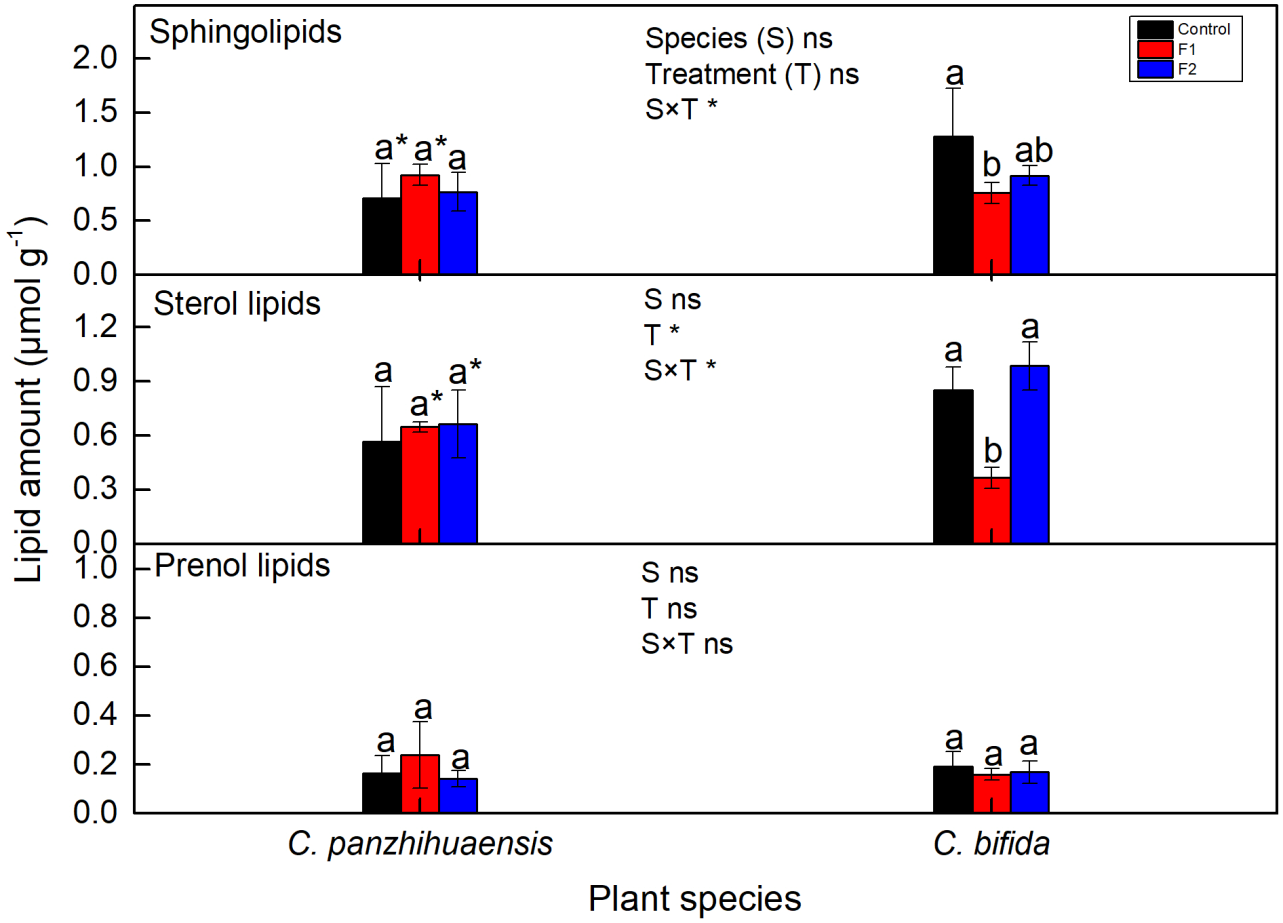
The content of sphingolipids, sterol lipids and prenol lipids of *Cycas panzhihuaensis* and *C. bifida* exposed to unexpected freezing stress. Two-way ANOVA analysis was performed in the general linear model. * indicates *P* ≤ 0.05. ns indicates not significant. For the same species, different letters in the same column are significantly different among treatments according to One-way ANOVA at P ≤ 0.05. For the same treatment, * is significantly different between species according to independent samples T-test at *P* ≤ 0.05. Values shown are the mean ± SD, *n* = 5. F1, freezing treatment at -5 °C for 1.5 h; F, freezing treatment at -5 °C for 6 h.

The amount of sphingolipids, sterol lipids, and prenol lipids remained unchanged in the freezing-treated *C. panzhihuaensis* (**Figure 8**). For *C. bifida*, the level of prenol lipids did not change but that of both sphingolipids and sterol lipids significantly declined in F1-treated plants but increased to the control level in F2-treated plants. Significant interaction was observed between species and treatment on the amounts of sphingolipids and sterol lipids.

## 4 Discussion

The maximum freezing tolerance of plants can be acquired by cold acclimation followed by freezing temperatures (Junttila and Kaurin, 1990; Herman et al., 2006). Some studies have investigated the effects of freezing temperatures following cold acclimation on the metabolic process of plants including carbohydrates, enzyme activity, amino acid, and lipids (Tan et al., 2018; Zareei et al., 2021) and found the metabolic adjustment of plants. However, how plants respond to unexpected freezing temperatures is not well understood.

Plants acquire carbon and energy through photosynthesis, which, however, is extremely sensitive to various stresses (Gao et al., 2020). Chlorophyll fluorescence is generally used to evaluate the photosynthetic activity under environmental stress (Hanachi et al., 2014; Kalaji et al., 2016). The maintenance and recovery of all the chlorophyll fluorescence parameters of *C. panzhihuaensis* (**Table 1**) suggest that the photosynthetic machinery of this species was stable when exposed to unpredicted freezing stress. This was consistent with the maintenance of the leaf morphological traits following recovery (**Supplementary Figure S1**). The dramatic decline of Fv/Fm, Y(II), qP, and rETR shows that the process of light reaction of photosynthesis in *C. bifida* was inhibited by freezing stress, being more severely with the extension of freezing time. Considering the partial or complete recovery of Fv/Fm, Y(II), qP, and rETR in F1-treated plants following 3 days of recovery, the photoinhibition induced by F1 treatment can be reversible. However, the further decrease of Fv/Fm, Y(II), qP, rETR and Y(NPQ) and the increase of Y(NO) in F2-treated plants after recovery suggest that *C. bifida* could not avoid photodamage of PSII induced by excessive light energy. On the basis of the yellowing of leaves in the recovered plants (**Supplementary Figure S1**), the photosynthetic disorders of F2-treated *C. bifida* were likely due to the injury of the photosynthetic apparatus that are related to overproduction of reactive oxygen species (ROS) in chloroplasts (Nishiyama et al., 2001). Low temperature has an immediate effect on enzymatic activities, and the chemical reaction rates are reduced by a factor of 2–3 per 10°C according to the van’t Hoff rule (Reyes et al., 2008; Seydel et al., 2022). Therefore, the reduced photosynthetic capacity might be also related to the freezing-induced lowered activities of enzymes involved in CO_2_ assimilation. Taken together, the photosynthetic machinery of *C. panzhihuaensis* exhibited higher tolerance to unpredicted freezing stress compared to *C. bifida*. Therefore, the unexpected freezing event will impose more negative effects on plant growth and survival of *C. bifida*. This demonstrates that *C. panzhihuaensis* possesses the potential to be introduced to the areas of higher latitudes and altitudes. However, the degree of its freezing tolerance needs to be ascertained.

Low temperatures can potentially disturb the energy balance through inhibiting photochemical processes and decreasing the rates of the enzymatic reactions (Huner et al., 1998; Ensminger et al., 2006). Energy charge is generally used to reflect the status of energy metabolism (Wang T. et al., 2020). The different change trend of energy charge between the two species (**Figure 1**) suggest that the two species adopted different strategies of energy metabolism under unexpected freezing stress that reflected the different cellular process and stress response. The respiration capacity of the alternative pathway will increase under adverse conditions to avoid the photodamage of photosystems (Zhang et al., 2012). Considering the stability of photosynthetic machinery in *C. panzhihuaensis*, the decrease of  energy charge with the prolongation of freezing treatment might be due to the increased utilization of energy in the stress defense or/and the enhanced photoprotection by mitochondrial alternative pathway. Considering the recovery of starch level and the accumulation of neutral glycerolipids following F2 treatment in *C. bifida* (**Figures 2**, **4**), the significant increase of energy charge might imply that the energy accumulated substantially in leaves. Niu et al. (2021) reported that the degree of postharvest yellowing of the tobacco leaves had a significant positive correlation with energy charge and the tobacco variety that is more prone to postharvest yellowing and browning maintained higher level of energy charge. However, the causal relationship between energy status and leaf damage and senescence under various environmental conditions needs to be explored. As only the leaves of F2-treated *C. bifida* cannot recover and ultimately died, whether the accumulation of energy charge can be used as a biomarker of freezing-induced severe damage should be further confirmed.

Carbohydrates are direct products of photosynthetic CO_2_ assimilation, and the tight regulation of carbohydrate and photosynthesis metabolism is needed to stabilize photosynthetic efficiency and energy metabolism (Seydel et al., 2022; Kitashova et al., 2023). The accumulation of soluble sugars is one of adaptive mechanisms adopted by some plants to cope with low temperatures (Yano et al., 2005; Thalmann and Santelia, 2017). However, the level of soluble sugars do not change or even decrease for some other plants subjected to low temperatures (Yildiz et al., 2014; Jiang et al., 2016; Ding et al., 2023). The research by Yildiz et al. (2014) and Zareei et al. (2021) suggest that the carbohydrate amount of some plant species/cultivars presents dynamic change with the increase of severity of freezing stress. Studies have shown that sugar phosphates accumulate at low temperatures, which can regulate carbon distribution between structural compounds, starch, and biosynthesis of soluble intermediates (Gray and Heath, 2005; Guy et al., 2008; Domon et al., 2013). For *C. panzhihuaensis*, considering the stability of photosynthetic machinery, significant decrease of the total soluble sugar level following F1 treatment (**Figure 2**) might be an defense response to cope with the sudden decrease of temperatures, and more soluble sugars might be converted to some other protective substances or structures. The stems of *Cycas* species are rich in starch, and their fleshy roots are rich in proteins, lipids, and carbohydrates (Lu, 2006). Whether the accumulated soluble sugars in F2-treated plants in comparison with that of the F1-treated plants were transferred from the fleshy roots and/or stems of *C. panzhihuaensis* needs to be verified. Although the soluble sugars recovered to the control level, sugar remodeling occurred in F2-treated plants of *C. panzhihuaensis* in comparison with the control group. Studies show that the contributing sugar in the freezing tolerance depends on plant species (Knaupp et al., 2011; Taïbi et al., 2018). Both sucrose and raffinose are non-reducing sugars, which can stabilize membranes (Strauss and Hauser, 1986; Nägele and Heyer, 2013). The significant accumulation of sucrose and raffinose might be conducive to the acquirement of tolerance to freezing stress in F2-treated plants of *C. panzhihuaensis*.

For *C. bifida*, the continuous decrease of soluble sugar level with the extension of freezing time was likely to be related to the increasing photoinhibition. The regulation of starch turnover is regulated by the rate of starch synthesis and degradation (Thalmann et al., 2016; Fernandez et al., 2017). Under adverse environmental conditions, starch is generally degraded to supply soluble sugars, energy, and other derived metabolites, which can mitigate the effects of stress (Thalmann and Santelia, 2017). The decreased starch level of *C. bifida* after F1 treatment might be due to the declined synthesis rate and/or enhanced degradation. However, the mechanism of the recovery of starch to the control level again after F2 treatment is not understood. It might be associated with a decreased sink capacity for photosynthate and relatively more soluble sugars converted to starch. In addition, cell wall polysaccharides can decrease in amount under low temperatures, and they can maintain energy homeostasis during senescence of green leaves serving as carbon source (Bilska-Kos et al., 2017; Biswal and Pandey, 2018). However, whether the recovery of starch in F2-treated *C. bifida* is related to the adjustment of cell wall polysaccharides is not sure. The continuous decline of NSC during freezing period might be an indicator of freezing sensitivity in *Cycas* species, which should be further confirmed.

Carbohydrates and lipids can interact in the modulation of carbon and energy homeostasis (Yu et al., 2018; Huang et al., 2019). Some studies suggest that more carbon flow is driven to lipid synthesis under some environmental conditions (Sun et al., 2016; Rai et al., 2017). As the amount of other lipid categories maintained unchanged, whether the significant accumulation of DAG in the F1-treated plants of *C. panzhihuaensis* (**Figure 4**) was associated with the depletion of the soluble sugars is not clear. DAG is an important intermediate in metabolism of both TAG and membrane lipids (Sergeeva et al., 2021). For *C. panzhihuaensis*, the transient accumulation of DAG at the early freezing stage (F1) might tend to provide signaling molecules, energy, and carbon, which enable plants to respond to subsequent stress rapidly. TAG is often accumulated in plants that are subjected to challenging environmental conditions (Gasulla et al., 2013; Higashi et al., 2015). For *C. bifida*, the levels of both DAG and TAG increased significantly after F2 treatment. In addition to the enhanced *de novo* synthesis, the metabolism of preexisting membrane lipids contributes to DAG formation and then TAG accumulation under various stress (Sun et al., 2016; Mueller et al., 2017; Rai et al., 2017; Young et al., 2022). The accumulated DAG and TAG after F2 treatment might be derived from the soluble sugars and/or membrane lipids that reduced concomitantly.

The membrane damage induced by stresses is generally accompanied with the degradation of membrane lipids (Zheng and Li, 2017; Wang Y. et al., 2020). Saccharolipids and PG are crucial for the maintenance of the photosynthetic machinery (Sakurai et al., 2003; Kobayashi et al., 2013). For *C. bifida*, the degradation of saccharolipids and phospholipids (**Figures 5**, **7**) following freezing treatments might be a key factor leading to photoinhibition. The origin of the accumulated membrane lipids in F2-treated *C. panzhihuaensis* was not determined. However, the accumulation of membrane glycerolipids might be conducive to stabilizing both the plastidic and extraplastidic membranes of *C. panzhihuaensis* under prolonged freezing temperatures.

Sphingolipids and sterol lipids can preferentially interact with each other and segregate to form microdomains that dubbed the membrane raft (Simons and Toomre, 2000). The levels and composition of sterols and sphingolipids can affect membrane stability, the activity of membrane proteins, and signal transduction processes (Lynch and Dunn, 2004; Sharfman et al., 2014; Yu et al., 2021). Considering the strong freezing tolerance of *C. panzhihuaensis*, the maintenance of the levels of sphingolipids and sterol lipids (**Figure 8**) might be associated with the membrane stability. The consistent change of sterol lipids and sphingolipids might reflect the dynamic change of membrane rafts during the freezing treatment in *C. bifida*. However, the specific roles of these changes are not clear and need to be further explored.

With the prolongation of freeing time, the amounts of most categories of the membrane lipids presented different change trend between *C. panzhihuaensis* and *C. bifida*, which might be related to their differential tolerance to unpredicted freezing stress.

## 5 Conclusions

The photosynthetic machinery of *C. panzhihuaensis* was more stable under the freezing stress in comparison with that of *C. bifida*. The energy charge showed a different change trend with the extension of freezing treatment time between *C. panzhihuaensis* and *C. bifida*. Moreover, with the prolongation of freezing time, the most components of NSC and most lipid categories and lipid classes changed differently in amount between the two species. These results suggest that the different freezing tolerance between the two species might be related to the differential patterns of the metabolism of energy and related substances. The continuous decrease of soluble sugars, the degradation of membrane glycerolipids, and the increment of neutral glycerolipids and energy charge might be used as predictors of freezing sensitivity in Cycas species, which should be further confirmed. The results show that *C. panzhihuaensis* can well cope with the unexpected freezing temperatures and possesses the potential to be introduced to the areas of higher latitudes and altitudes. However, several questions still remain to be answered, so further work will be undertaken to explore the dynamic change of physiological processes including photosynthesis, respiration, and photorespiration; the cooperation of different organs in carbon metabolism; and the molecular mechanisms underlying the changes of energy and related substances under unexpected freezing stress.

## Data availability statement

The original contributions presented in the study are included in the article/**Supplementary Material**. Further inquiries can be directed to the corresponding author.

## Author contributions

YW: Data curation, Formal analysis, Writing – review & editing. HZ: Data curation, Formal analysis, Writing – review & editing. ZL: Formal analysis, Writing – review & editing. EL: Formal analysis, Writing – review & editing. XP: Writing – review & editing. YZ: Formal analysis, Funding acquisition, Writing – original draft, Conceptualization.
