# Supplementary material for: The metabolism of nonstructural carbohydrates, lipids, and energy in two Cycas species with differential tolerance to unexpected freezing stress
Source: Front Plant Sci. 2023 Dec 7;14:1301560. doi: 10.3389/fpls.2023.1301560 (PMC10740210; doi:10.3389/fpls.2023.1301560)
Supplement: Supplementary file 1 [file DataSheet_1.doc]

**Supplementary figure**


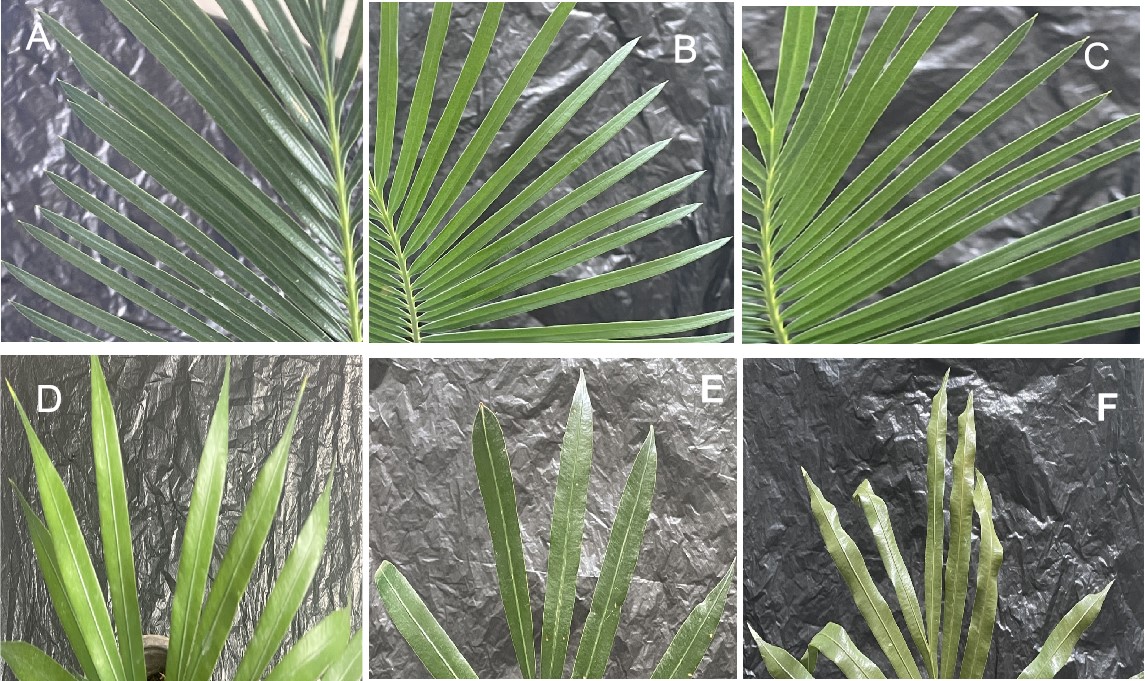


Fig. S1 The leaf morphological characteristics of freezing-treated *Cycas panzhihuaensis* and *C. bifida* following 3 d of recovery under control conditions (25/15 °C). A, B, and C are the plant leaves of *C. panzhihuaensis* previously subjected to control, F1 (exposure of plants to -5 °C for 1.5 h) and F2 (exposure of plants to -5 °C for 6 h) treatment, respectively; D, E and F are the plant leaves of *C. bifida* previously subjected to control, F1 and F2 treatment, respectively.
